# Predictors of anxiety in endometriosis patients

**DOI:** 10.1007/s00404-024-07878-4

**Published:** 2024-12-27

**Authors:** Tomas Kupec, Lisa Wagels, Rebecca Caspers, Philipp Meyer-Wilmes, Laila Najjari, Elmar Stickeler, Julia Wittenborn

**Affiliations:** 1 Department of Gynecology and Obstetrics, University Hospital of the RWTH Aachen, Pauwelsstrasse 30, 52074 Aachen, Germany; 2 Department of Psychiatry, Psychotherapy and Psychosomatics, University Hospital of the RWTH Aachen, Pauwelsstrasse 30, 52074 Aachen, Germany; 3 Research Center Jülich, JARA Institute Brain Structure Function Relationship Institute for Neuroscience and Medicine (INM-10), 52425 Jülich, Germany

**Keywords:** Endometriosis, Anxiety, STAI, Lower abdominal pain

## Abstract

**Purpose:**

To evaluate the main factors influencing anxiety in endometriosis patients presenting to an endometriosis centre in Germany.

**Methods:**

One hundred and eighty-two patients were asked to complete the German version of the STAI (state anxiety and trait anxiety) questionnaire prior to examination for diagnosis and treatment of pelvic pain or suspected endometriosis. Typical endometriosis symptoms, main complaints, operations, type of endometriosis and planned treatment were analyzed as influencing factors of anxiety in endometriosis patients. We performed linear multiple regression analyses using the forward stepwise method to test which characteristics associated with endometriosis symptoms were associated with trait anxiety and state anxiety.

**Results:**

Analysis of the STAI results showed that higher levels of trait anxiety were found in patients with ovarian endometriosis: *t* (177) = 3.06, *p* = 0.003 and in patients with symptoms of dyspareunia: *t* (177) = 2.36, *p* < 0.020). On the other hand, patients with recurrent endometriosis showed lower levels of trait anxiety: *t* (177) = − 2.39, *p* = 0.018. Significantly higher levels of state anxiety were found in patients with persistent endometriosis: *t* (177) = − 2.45, *p* = 0.015 and in women with endometriosis who were indicated for surgical therapy: *t* (177) = 3.89, *p* < 0.001.

**Conclusions:**

We were able to show that higher levels of ongoing anxiety in endometriosis patients are associated with dyspareunia and ovarian endometriosis, which may have a negative impact on partnership and desire to have children. On the other hand, patients with persistent endometriosis or a type of disease that requires surgery have higher levels of immediate situational anxiety.

**Supplementary Information:**

The online version contains supplementary material available at 10.1007/s00404-024-07878-4.

## What does this study adds to the clinical work

**Table:** 

We have investigated the relationship between endometriosis and anxiety and examined the main factors influencing anxiety in patients with endometriosis. We found correlating factors that influence anxiety as a state or as a personality trait in these patients. These findings provide valuable information about anxiety in women with endometriosis, which may help to improve their care.	

## Introduction

Endometriosis is a chronic gynaecological disorder characterized by the presence and growth of endometrial-like tissue outside the uterus. This ectopic tissue, often found on pelvic structures such as the ovaries, fallopian tubes and peritoneum, responds to hormonal fluctuations during the menstrual cycle. As a result, it undergoes cyclical changes, including inflammation, bleeding, and the formation of adhesions and scar tissue [1].

The main problem with endometriosis is the length of time between the onset of symptoms and the correct diagnosis. On average, it takes 10 years for endometriosis to be diagnosed. It is often only diagnosed as part of an infertility investigation [2]. A second problem is repeated surgeries in the chronic form of the disease, which usually would not be necessary, if the first surgery for endometriosis had been performed correctly [3].

Diagnosis often involves clinical assessment by gynaecological examination, vaginal ultrasound and medical history. Previous recommendations suggested laparoscopy as the gold standard for the treatment especially the diagnosis of endometriosis. The current ESHRE guideline of 2022 [4] has relativized this dogma. In most cases, anamnesis, gynaecological examination and vaginal sonography are sufficient for the diagnosis and to initiate multimodal treatment of endometriosis. The indication for a laparoscopy should be highly restricted as it entails an invasive surgical procedure under general anesthesia with associated risks and morbidity [4].

Endometriosis is a serious social problem due to its high incidence in young women. Approximately 10% of women of reproductive age may have endometriosis [5]. These findings are underestimated due to the high number of undiagnosed cases. At present, the disease is increasingly in the focus of interest. Still, the connection between endometriosis and psychological stress is often neglected in gynaecological practice. Due to the symptoms, such as pelvic pain, dysmenorrhea, dyspareunia, dysuria and dyschezia, the disease is often associated with absences from work, difficulties in having children, unnecessary operations and recurrences. The complexity of the disease leads not only to physical but also psychological stress. It has been shown that endometriosis is associated with an increased incidence of self-reported depression, anxiety and eating disorders [6, 7].

Endometriosis may negatively influence mental health through several pathways. Chronic pain can lead to social isolation and negatively affect emotional well-being [8–11]. It is known, from animal studies, that endometriosis alters gene expression and electrophysiology in the brain, leading to an increase in pain sensitization, anxiety and depression [12]. Finally, the chronic inflammation of endometriosis may affect the blood–brain barrier and disrupt certain areas of the brain, leading to mood or behavioral disorders [13–18].

A recently published systematic review supports the hypothesis that symptoms of depression and anxiety are common in people with endometriosis and are associated with chronic pain [19]. The authors recommend that the correlating factors should be investigated further, because it is largely unknown which factors associated with endometriosis increase the risk for anxiety problems. Notably, data concerning the association of anxiety problems and endometriosis across 28 reviewed articles ranged from 11.5 to 87.5% [20]. Especially, this huge range supports the assumption that factors related to the heterogeneity of the disease itself may mediate the prevalence of anxiety problems together with the disease.

Thus, this study aims to find the key influencing factors of endometriosis patients’ anxiety. As possible variables a wide range of endometriosis symptoms, previous endometriosis treatment and different therapy plans were evaluated. We present the evaluation of anxiety using the well validated state–trait-anxiety questionnaire (STAI) in patients attending our endometriosis centre for diagnosis and treatment of this disease.

## Materials and methods

### Sample

Data from a total of 182 patients who presented as outpatients with lower abdominal pain or suspected endometriosis to the Endometriosis Centre of the RWTH Aachen University Hospital between October 2022 and December 2023 were analyzed.

### Procedure

Before the examination, patients completed a standardized medical history form in the waiting room, including the German version of the STAI questionnaire (state anxiety and trait anxiety). Endometriosis was diagnosed and localized according to current ESHRE guidelines [4] after a gynaecological examination and ultrasound by a specialized consultant with many years of experience at the endometriosis centre. After the examination, a detailed medical consultation took place, during which the doctor and the patient discussed further treatment planning, as well as a counselling and support session on the topic of endometriosis.

### Materials

Demographic data and self-reported symptoms were recorded in standardized patient history form and during a medical interview with the patients. Clinical data were recorded in detail in a medical examination report. Pain severity, typical endometriosis symptoms, main complaints, operations, endometriosis diagnosis and planned procedures were assessed in all patients.

In addition, all patients filled in the German version of the STAI [21]. The STAI is a widely used questionnaire in research and clinical settings to assess anxiety levels and distinguish between temporary, situation-specific anxiety (state anxiety) and more enduring, personality-based anxiety (trait anxiety). It can be useful in several fields, including psychology, psychiatry, and medicine, to better understand and treat anxiety-related problems in individuals [21]. This self-report instrument has two scales of which the first assesses trait anxiety, defined as a relatively stable tendency to view situations as threatening and to react to this with an increase in state anxiety. The second scale assesses state anxiety, which is considered, and affective state characterized by high tension, anxiety, nervousness, inner restlessness and fear of future events. It is associated with increased activity of the autonomic nervous system. The scales consist of 20 items, respectively, which are answered on a 4-point Likert-type scale. The scale has ten reverse-scored items.

### Statistical analyses

We performed linear multiple regression analyses applying the forward stepwise method to test which characteristics associated with endometriosis symptoms are associated to trait anxiety (model 1) and state anxiety (model 2). For the final models, we report the effect sizes that include predictors with significant contributions to the outcome measures. For statistical analyses, we used IBM SPSS Statistics for Windows, version 25.0.

## Results

The stepwise regression analysis suggested to retain only 3 of 27 variables for the trait-anxiety model. For an overview of the excluded variables see supplementary Table 1. We report the model statistics and the effect sizes for the variables included in the final model. Regarding trait anxiety (model 1), the model explaining most of the variance included the variables ovarian location of endometriosis (ovary), recurrence and dyspareunia (*R* = 0.307, *R*^2^ = 0.094, corrected *R*^2^ = 0.079, SD = 11.23, *F* (3,177) = 6.15, *p* < 0.001), which all contributed significantly to the model [ovary: *t* (177) = 3.06, *p* = 0.003; recurrence: *t* (177) = − 2.39, *p* = 0.018; dyspareunia: *t* (177) = 2.36, *p* < 0.020].

The stepwise regression analysis for the state-anxiety model suggested to retain two variables of 27 for the state-anxiety model. For an overview of the excluded variables see supplementary Table 2. Again, we report the model statistics and the effect sizes for the variables included in the final model. Regarding state anxiety (model 2), we found the model explaining most of the variance included the variables surgical therapy and persistent endometriosis (*R* = 0.307, *R*^2^ = 0.095, corrected *R*^2^ = 0.084, SD = 10.70, *F* (2177) = 9.34, *p* < 0.001), which contributed significantly to the model [surgical therapy: *t* (177) = 3.89, *p* < 0.001; persistent endometriosis: *t* (177) = 2.45, *p* = 0.015].

**Fig. 1:**
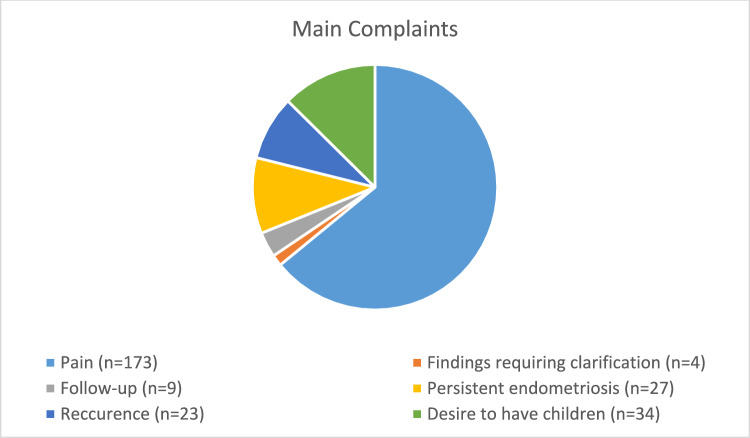
Reason for the presentation of the patients to the Endometriosis Centre of the RWTH Aachen University Hospital

The main reasons why patients presented to the Endometriosis Centre of the RWTH Aachen University Hospital are shown in Fig. 1. Some of the patients had more than one main complaint, which were subjectively of equal importance. General descriptive statistics about clinical data and reported symptoms of the patients are given in Table 1.

**Table 1:** Patient characteristics

	Mean (SD)
Age	27.9 (8.76)
Pain severity (visual analogue scale)	7.26 (1.92)
Parity	0.24 (0.60)
STAI-state	48 (11.72)
STAI-trait	45 (11.18)
Number of patients *n* (%)	
First diagnosis	108 (59.3)
Previous therapy	74 (40.7)
Current therapy	
None	103 (56.6)
Endocrine therapy	67 (36.8)
Analgesia	12 (6.6)
Typical endometriosis symptoms	
Dysmenorrhoea	176 (96.7)
Dyspareunia	69 (37.9)
Dysuria	19 (10.4)
Dyschezia	35 (19.2)
Main complaints	
Pain	173 (95.0)
Sterility	7 (3.8)
Findings requiring clarification	4 (2.2)
Follow-up	9 (4.9)
Persistent endometriosis	27 (14.8)
Recurrence	23 (12.6)
Desire to have children	34 (18.7)
Operations	
Previous abdominal surgery	126 (69.2)
Previous endometriosis surgery	83 (45.6)
Previous histological confirmation of endometriosis	64 (35.2)
Location of endometriosis diagnosis	
Peritoneal	115 (63.2)
Ovary	10 (5.5)
Deep infiltrating endometriosis	20 (10.9)
Adenomyosis Uteri	80 (43.9)
Planned procedure	
Surgical therapy	64 (35.2)
Drug-based pain therapy	17 (9.3)
Multimodal pain therapy	171 (93.9)
Reproductive medicine	6 (3.3)
Endocrine therapy	131 (71.9)
Complementary procedure	177 (97.3)

## Discussion

The aim of this study was to investigate the relationship between endometriosis and anxiety and to examine key influencing factors of anxiety in patients attending our endometriosis centre. We found different correlating factors focusing on state anxiety or anxiety as a personality trait.

State anxiety was associated with persistent endometriosis and surgical therapy as the planned procedure. Patients with persistent endometriosis and those who are scheduled for surgery had higher levels of currently indicated anxiety. We suggest that there could be a direct link between state anxiety and the uncertainty or fear of the consequences of the specifically planned procedure.

In contrast, trait anxiety was not associated with planned procedures. Instead, patients presenting to our centre with a diagnosis of ovarian endometriosis and symptoms of dyspareunia had higher levels of trait anxiety, whereas patients with recurrent endometriosis and those who came for diagnosis and treatment of recurrent endometriosis symptoms had lower levels of trait anxiety.

Ovarian endometriosis is not only associated with symptoms typical of endometriosis, but also with the loss of follicles. For this reason, recurrence operations should be avoided as far as possible in order to protect the follicles. However, this is not always possible. Infertility or subfertility and concerns about potential infertility are a huge source of worry or depression [22, 23]. Especially, since has been proven that AMH (anti-Müllerian hormone) decreases after every ovarian operation [24]. Patients with ovarian endometriosis may have higher STAI-Trait scores due to anxiety about their fertility. Indirect support for this assumption is given by a study showing, that women with endometriosis who had previously been pregnant had lower rates of anxiety [25].

Dyspareunia is an important symptom of endometriosis [26], affecting up to 79% of young women with endometriosis, including adolescents [27]. This symptom has a negative impact on partnership and quality of life in general. Patients with dyspareunia are also more likely to experience pain during gynaecological examinations [28]. These findings, particularly the anxiety associated with dyspareunia may explain the increased STAI-Trait scores seen in this study. Facchin F. et al. [29] support our findings, that dyspareunia is associated with increased symptoms of anxiety, using in his study the HADS-A (Hospital Anxiety and Depression Scale-Anxiety) to examine anxiety levels in endometriosis patients.

Patients with lower trait anxiety had more often recurrent endometriosis. Endometriosis recurs in up to 18–43% of cases [30, 31] and the likelihood of recurrence increases with time after primary surgical therapy. The risk of endometriosis recurrence is significantly lower in patients who received postoperative hormonal suppression compared with expectant management or placebo [32]. To achieve the lowest recurrence rate, adjuvant treatment with dienogest should last at least 24 months [33]. In patients with endometriosis after 1 year of treatment with Dienogest and a significant improvement in pain, a reduction in anxiety was demonstrated [34]. Patients in this study had already been successfully treated with the previous therapy until recurrence. Thus, lower anxiety can be explained by the expectation of further successful therapy.

Patients with persistent endometriosis (*n* = 27) who presented to our clinic had already been treated for endometriosis in the past, including surgical removal of the endometriosis. However, the previous treatment had not been successful. Unfortunately, only about 40% of patients with endometriosis are treated in such specialized facilities. As a result, patients often do not receive treatment according to current guidelines and surgical treatment of endometriosis is incomplete, often leading to unnecessary re-operations. This lack of optimal treatment often leads to frustration and chronic pain in endometriosis patients [35]. Rates of re-operation after endometriosis surgery have been reported to be between 27 and 58% [36–38]. The previous unsuccessful, mostly repeated threat of the disease, and probably the fear that the new treatment in our department would also fail, was reflected in increased STAI-State scores, as shown in this study.

Patients (*n* = 64) who were recommended for surgical treatment at our centre had higher state anxiety. Almost 2/3 of these patients have already undergone previous therapy without improvement of symptoms. High anxiety may reflect an increased fear of ineffective treatment. Moreover, surgery can be a cause for increased anxiety. Other investigations show, that among surgical patients, almost 48% had a prevalence of preoperative anxiety [39].

The average age of the patients in this study was 27.9 years, which is lower than in the large studies already published [40, 41]. We attribute the younger average age to the fact that the gynaecologist in the practice and the patient in our region are well informed and the patients come to the endometriosis centre earlier than usual.

The implication of this study for clinical practice in the diagnosis and management of patients with endometriosis is that patients with dyspareunia, ovarian endometriosis, persistent endometriosis and women who require surgical endometriosis management are more at risk of anxiety and should be given more attention by the gynaecologist during the consultation and should possibly be offered an appointment with a psychologist/psychiatrist.

A wide range of possible influencing factors of endometriosis patients’ anxiety were included in this study, covering all important areas of clinical practice in endometriosis in a large, specialized patient group. But of course, we cannot claim that all possible influencing factors were complete. Another limitation of this study was that it was a monocentric study.

The strength of this study is that we included in our analysis model all the variables that are important in the history, complaints, examination and planning of multimodal therapy in patients with endometriosis. All patients were examined in the certified endometriosis centre. The data collected were analyzed and the examination was carried out by a senior consultant with many years of experience in an endometriosis centre.

In conclusion, we can show that higher levels of anxiety as trait characteristic in endometriosis patients is associated with dyspareunia and ovarian endometriosis, which may have a negative impact on partnership and desire to have children. Acute anxiety seems to have different sources. Patients with persistent endometriosis or a type of disease that requires surgery have higher levels of immediate situational anxiety.

## Supplementary Information

Below is the link to the electronic supplementary material.

Supplementary file1 (DOCX 15 KB)

Supplementary file2 (DOCX 15 KB)

## Data Availability

The datasets generated during the current study are available from the corresponding author on reasonable request.
