# Supplementary material for: Predictors of anxiety in endometriosis patients
Source: Arch Gynecol Obstet. 2024 Dec 27;311(5):1371–7. doi: 10.1007/s00404-024-07878-4 (PMC12033099; doi:10.1007/s00404-024-07878-4)
Supplement: Supplementary file 1 — Supplementary file1 (DOCX 15 KB) [file 404_2024_7878_MOESM1_ESM.docx]

**Predictors of anxiety in endometriosis patients**

**Archives of Gynecology and Obstetrics**

Tomas Kupec^1,2^, Lisa Wagels, Rebecca Caspers, Philipp Meyer-Wilmes, Laila Najjari, Elmar Stickeler, Julia Wittenborn

^1^Department of Gynecology and Obstetrics, University Hospital of the RWTH Aachen, Pauwelsstrasse 30, 52074, Aachen, Germany.

^2^Corresponding author: Tomas Kupec, [tkupec@ukaachen.de](mailto:tkupec@ukaachen.de)

**Supplementary table 1:** Summary on excluded variables for the regression model on trait anxiety (Stepwise forward regression).

|  | Beta | T | Sig | Partial correlation | Collinearity |
| --- | --- | --- | --- | --- | --- |
| Age | .044 | .584 | .560 | .044 | .926 |
| Pain severity (Visual analogue scale) | .076 | 1.024 | .307 | .077 | .939 |
|  |  |  |  |  |  |
| **Typical endometriosis symptoms** |  |  |  |  |  |
| Dysmenorrhoea | .004 | .056 | .956 | .004 | .962 |
| Dysuria | .045 | .616 | .539 | .046 | .950 |
| Dyschezia | -.083 | -1.151 | .251 | -.086 | .986 |
|  |  |  |  |  |  |
| **Main complaints** |  |  |  |  |  |
| Pain | .025 | .341 | .733 | .026 | .956 |
| Sterility | .122 | 1.697 | .092 | .127 | .984 |
| Findings requiring clarification | -.034 | -.472 | .638 | -.036 | .982 |
| Follow-up | -.140 | -1.960 | .052 | -.146 | .984 |
| Persistent endometriosis | .111 | 1.555 | .122 | .116 | .989 |
| Desire to have children | .068 | .951 | .343 | .071 | .998 |
|  |  |  |  |  |  |
| **Operations** |  |  |  |  |  |
| Previous abdominal surgery | .003 | .034 | .973 | .003 | .807 |
| Previous endometriosis surgery | .001 | .010 | .992 | .001 | .761 |
| Previous histological confirmation of endometriosis | .073 | .914 | .362 | .069 | .803 |
|  |  |  |  |  |  |
| **Location of endometriosis diagnosis** |  |  |  |  |  |
| Peritoneal | .097 | 1.344 | .181 | .101 | .975 |
| Deep infiltrating | -.078 | -1.056 | .293 | -.079 | .944 |
| Adenomyosis Uteri | -.003 | -.042 | .966 | -.003 | .973 |
|  |  |  |  |  |  |
| **Planned procedure** |  |  |  |  |  |
| Surgical therapy | .111 | 1.474 | .142 | .110 | .895 |
| Drug-based pain therapy | .120 | 1.680 | .095 | .126 | .994 |
| Multimodal pain therapy | -.109 | -1.514 | .132 | -.113 | .973 |
| Reproductive medicine | .092 | 1.274 | .204 | .096 | .976 |
| Analgesia | .014 | .187 | .852 | .014 | .978 |
| Endocrine therapy | -.037 | -.510 | .610 | -.038 | .969 |
| Complementary procedure | -.055 | -.744 | .458 | -.056 | .935 |
|  |  |  |  |  |  |
|  |  |  |  |  |  |
