# Supplementary material for: Predictors of anxiety in endometriosis patients
Source: Arch Gynecol Obstet. 2024 Dec 27;311(5):1371–7. doi: 10.1007/s00404-024-07878-4 (PMC12033099; doi:10.1007/s00404-024-07878-4)
Supplement: Supplementary file 2 — Supplementary file2 (DOCX 15 KB) [file 404_2024_7878_MOESM2_ESM.docx]

**Predictors of anxiety in endometriosis patients**

**Archives of Gynecology and Obstetrics**

Tomas Kupec^1,2^, Lisa Wagels, Rebecca Caspers, Philipp Meyer-Wilmes, Laila Najjari, Elmar Stickeler, Julia Wittenborn

^1^Department of Gynecology and Obstetrics, University Hospital of the RWTH Aachen, Pauwelsstrasse 30, 52074, Aachen, Germany.

^2^Corresponding author: Tomas Kupec, [tkupec@ukaachen.de](mailto:tkupec@ukaachen.de)

**Supplementary table 2:** Summary on excluded variables for the regression model on trait anxiety (Stepwise forward regression).

|  | Beta | T | Sig | Partial correlation | Collinearity |
| --- | --- | --- | --- | --- | --- |
| Age | -.114 | -1.591 | .113 | -.119 | .988 |
| Pain severity (Visual analogue scale) | .075 | 1.025 | .307 | .077 | .962 |
|  |  |  |  |  |  |
| **Typical endometriosis symptoms** |  |  |  |  |  |
| Dysmenorrhoea | .023 | .327 | .744 | .025 | .995 |
| Dyspareunia | .076 | 1.014 | .312 | .076 | .905 |
| Dysuria | .126 | 1.726 | .086 | .129 | .947 |
| Dyschezia | -.070 | -.963 | .337 | -.072 | .976 |
|  |  |  |  |  |  |
| **Main complaints** |  |  |  |  |  |
| Pain | .056 | .775 | .439 | .058 | .990 |
| Sterility | .013 | .172 | .864 | .013 | .952 |
| Findings requiring clarification | -.034 | -.474 | .636 | -.036 | .973 |
| Follow-up | -.074 | -1.019 | .309 | -.077 | .965 |
| Reccurence | -.108 | -1.514 | .132 | -.113 | .995 |
| Desire to have children | -.041 | -.572 | .568 | -.043 | .985 |
|  |  |  |  |  |  |
| **Operations** |  |  |  |  |  |
| Previous abdominal surgery | -.130 | -1.690 | .093 | -.126 | .850 |
| Previous endometriosis surgery | -.118 | -1.483 | .140 | -.111 | .800 |
| Previous histological confirmation of endometriosis | -.082 | -.978 | .329 | -.074 | .724 |
|  |  |  |  |  |  |
| **Location of endometriosis diagnosis** |  |  |  |  |  |
| Peritoneal | .114 | 1.537 | .126 | .115 | .927 |
| Ovary | .109 | 1.515 | .132 | .113 | .978 |
| Deep infiltrating | -.097 | -1.350 | .179 | -.101 | .988 |
| Adenomyosis Uteri | -.106 | -1.471 | .143 | -.110 | .979 |
|  |  |  |  |  |  |
| **Planned procedure** |  |  |  |  |  |
| Drug-based pain therapy | .044 | .610 | .543 | .046 | .978 |
| Multimodal pain therapy | -.023 | -.318 | .751 | -.024 | .989 |
| Reproductive medicine | .068 | .954 | .341 | .072 | .999 |
| Analgesia | .092 | 1.292 | .198 | .097 | .998 |
| Endocrine therapy | -.108 | -1.497 | .136 | -.112 | .969 |
| Complementary procedure | .003 | .045 | .964 | .003 | .973 |
|  |  |  |  |  |  |
|  |  |  |  |  |  |
